# Epidemiological characteristics, clinical characteristics, and prognostic factors of children with atopy hospitalised with adenovirus pneumonia

**DOI:** 10.1186/s12879-021-06741-0

**Published:** 2021-10-09

**Authors:** Miao Li, Xiao-Hua Han, Li-Yun Liu, Hui-Sheng Yao, Li-Li Yi

**Affiliations:** Department of Pediatrics, Shengjing Hospital of China Medical University, Shenyang, 110004 Liaoning China

**Keywords:** Adenoviral infection, Atopy, Children, Small airway lesions

## Abstract

**Background:**

Atopy may be associated with disease severity and a poor prognosis of human adenovirus (HAdV) pneumonia in children. Our aim was to observe the clinical characteristics and pulmonary radiological changes in children with atopy and HAdV pneumonia in China.

**Methods:**

Children hospitalised with HAdV pneumonia from June 2018 to December 2019 were analysed. All children were divided into atopic with HAdV, non-atopic with HAdV, and atopic without HAdV infection group. Each group was further divided into the mild and severe pneumonia groups according to disease severity. Standard treatment was initiated after admission, and regular follow-up evaluations were conducted at 1 month after discharge. Baseline and clinical characteristics and pulmonary radiological changes in children with and without atopy were evaluated. Risk factors associated with small airway lesions in patients with HAdV pneumonia were analysed.

**Results:**

The eosinophil count in the atopic group was significantly higher than that in the non-atopic group (*P* < 0.05). Severe coughing, wheezing, and small airway lesions on chest high-resolution computed tomography (HRCT) upon admission, after discharge and 1 month after discharge were significantly higher in the atopic group (with or without HAdV infection) than in the non-atopic group (*P* < 0.05). There were significant differences in the number of patients with wheezing and small airway lesions during hospitalisation and after discharge among the three groups (*P* < 0.05). The risks of small airway lesions in children with a family or personal history of asthma, severe infection, atopy, and HAdV infection were 2.1-, 2.7-, 1.9-, 2.1-, and 1.4-times higher than those in children without these characteristics, respectively.

**Conclusions:**

Children with atopy and HAdV pneumonia may experience severe coughing in mild cases and wheezing in mild and severe cases. Children with atopy are more susceptible to the development of small airway lesions, recurrent wheezing after discharge and slower recovery of small airway lesions as observed on pulmonary imaging than non-atopic children after HAdV infection. A family or personal history of asthma, atopy, severe infection, and HAdV infection are independent risk factors associated with the development of small airway lesion as observed on chest HRCT.

## Background

Human adenovirus (HAdV) infection is a major cause of community-acquired pneumonia in infants and young children [1, 2]. HAdV pneumonia generally occurs in patients between 6 months and 5 years of age and especially affects children < 2 years of age. Nearly 5% of children infected with HAdV develop pneumonia, and nearly 1.3 million children die annually due to HAdV infection [3, 4]. The mortality rate of severe untreated pneumonia or disseminated disease caused by HAdV may exceed 50% [5, 6]. Children with severe HAdV pneumonia often require hospitalisation due to more severe clinical symptom and extra-pulmonary complications. Some patients also develop chronic airway and lung diseases that may lead to death [7]. Despite continuous advancements in medical technology and increasing administration of standardised HAdV treatment, some children still have a poor prognosis; thus, HAdV infection continues to require further attention. After infection, up to 30% of children have long-term respiratory complications such as post-infection bronchiolitis obliterans [8] and bronchiectasis [9], as well as an irreversible decline in lung function [10]. Therefore, understanding the prognosis of children with HAdV infection is the focus of the current research. We are particularly addressing the question of why some children experience severe symptoms and have a poor prognosis. In recent years, most clinical studies have focused on virus typing [11–13], but few studies have assessed the risk factors associated with a poor prognosis. Previous regional studies in Singapore have shown that an HAdV type 7 infection, a severe infection requiring invasive or non-invasive ventilation, and a family or personal history of asthma were risk factors for respiratory complications [14].

In children, atopy usually manifests as atopic dermatitis, allergic rhinitis, and allergic asthma; however, some children experience airway hyperresponsiveness [15]. Allergic inflammation may affect immunity; thus, children with atopy may be prone to recurrent respiratory tract infections [16], and some children may experience sequelae [14]. Many atopic children with HAdV infection were found experience wheezing and slower recovery than non-atopic children, and requiring long-term atomisation compared to previously healthy children. So we suppose that atopy may be related to the severity of pneumonia and poor prognosis of HAdV infection. In our study we aimed to analyse the baseline and clinical characteristics, and pulmonary radiological changes in children with HAdV infection, with and without atopy, to establish whether atopy is the one of the risk factors for small airway lesions observed during high-resolution computed tomography (HRCT) after HAdV infection. The early identification of risk factors is particularly important to remind clinicians to implement intervention strategies earlier and improve patient prognosis.

## Methods

### Study population

A total of 200 children were included: 120 children who were hospitalised with HAdV pneumonia from June 2018 to December 2019 at the Pediatric Respiratory Department of Shengjing Hospital. Because we wanted to study whether adenovirus infection could affect the severity of pneumonia and whether it could affect the prognosis of pneumonia, and want to analyze the reason for the poor prognosis of pneumonia is severity of pneumonia or atopy, 80 children with atopic and without HAdV infection were select as control group. Since the severity of pneumonia will affect the prognosis of pneumonia, we divided each group into severe and mild group respectively. We included children with pneumonia between 1 month and 14 years of age. Children with congenital pulmonary dysplasia, airway malformation, congenital immune deficiency disease, congenital heart disease, malnutrition, or congenital metabolic diseases were excluded. Our study is a retrospective study, and the data statistics are anonymous, the need for patient consent was waived by the ethics committee (No: 2020PS005K).

### Study definitions

Pneumonia was diagnosed based on the patient’s symptoms (fever, cough, and/or rapid breathing), clinical evidence (tachypnoea, chest recessions, and/or adventitious sounds upon lung auscultation), and radiographic signs (infiltrates or consolidation). Severe HAdV pneumonia was defined as that requiring either invasive or non-invasive respiratory support, requiring paediatric intensive care unit care, or resulting in death. Atopy was diagnosed by a specialist based on the patient’s history of atopic dermatitis, asthma, and allergic rhinitis, as well as results from quantitative allergen-specific IgE and skin scratch tests.

### Data collection

The medical records of the children were retrospectively reviewed and analysed to extract data on the following factors: age; sex; duration of fever; duration of hospitalisation; laboratory test results at the first admission (neutrophil count, eosinophil count, lymphocyte count, and C-reactive protein [CRP] level); the presence of dyspnoea, extra-pulmonary complications, and/or wheezing upon admission, after discharge, and 1 month post-discharge; the presence of severe coughing (nocturnal cough or vomiting after cough); chest CT or X-ray results (children with wheezing underwent HRCT), and the presence of small airway lesions on chest HRCT images. Two specialists in chest radiography independently reviewed the scans. Data on baseline and clinical characteristics as well as pulmonary radiological changes in children with and without atopy were collected. Risk factors associated with post-HAdV pneumonia and small airway lesions were analysed.

In the children with atopy, quantitative allergen-specific IgE and skin scratch tests were also performed within 24 h if the children had not undergone an allergen-specific IgE test within the previous 6 months. If specific IgE levels exceeded 0.35 kUa/L (Allergen-specific IgE testing kit; Semel Technologies Ltd., Shenyang, China), exposure to any allergens was avoided.

### Specimen collection and virus identification

Nasopharyngeal swabs for the identification of HAdV antigens (Respiratory Virus detection kit; Shanghai Xing Yao Medicine Technology Development Co. Ltd., Shanghai, China) and HAdV DNA (HAdV RNA detection kit; Shenzhen PuRuiKang Biological Technology Co. Ltd., Shenzhen, China) were collected within 24 h after admission.

### Treatment modalities

The diagnosis and treatment of HAdV infection were performed according to the national guidelines published in 2019 [17]. All children received standard treatment for pneumonia, which included supportive care when necessary, invasive or non-invasive ventilation, and antibiotics. As gamma globulin has the role of enhance IgG function, inhibiting and neutralizing inflammatory factors, and neutralizing viruses, it was administered intravenously to children with persistent fever, while intravenous pulse methylprednisolone was administered to children with severe disease, including rapidly increasing respiratory distress, at the discretion of the treating physician. Children with wheezing were treated with a bronchodilator to relax the airway. Children who remained non-febrile for longer than 48 h, had no wheezing, experienced relief from cough, and showed a 50% decrease in infiltrates or the consolidation area (compared to the values at admission) were discharged from the hospital. For children who experienced wheezing or showed abnormalities on their chest HRCT image, a follow-up visit was scheduled for 1 month after discharge to observe whether wheezing remained and to assess any HRCT image changes. If the chest CT findings normalized before discharge, no re-examination was required.

### Statistical analysis

Baseline and clinical characteristics as well as pulmonary radiological changes in children with and without atopy were evaluated. The risk factors associated with small airway lesions in patients with HAdV pneumonia were analysed. Continuous variables are described as the mean ± standard deviation. A one-way analysis of variance (ANOVA) was used to assess the differences among the three groups, and the Student–Newman–Keuls or least significant difference test was used as the post hoc test. The chi-square test was applied for categorical variables, and multivariate analysis was used to identify risk factors associated with small airway lesions in patients with HAdV pneumonia. Spearman’s test was used to assess correlation. All statistical analyses were conducted using SPSS 20.0. Two-sided P values < 0.05 were considered statistically significant.

## Results

**Fig. 1:**
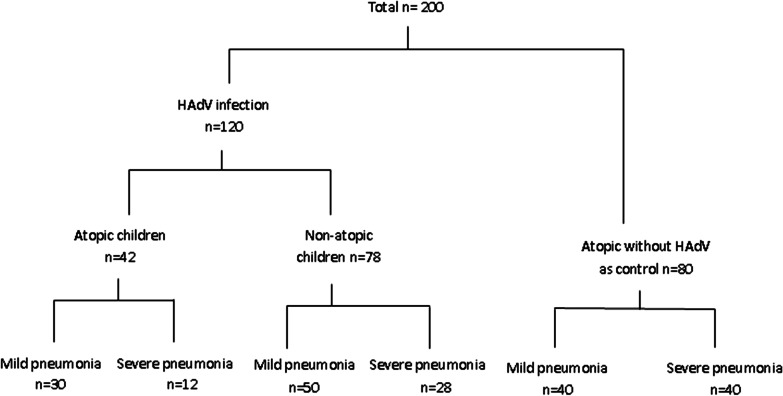
Chart for patient grouping. A total of 200 children were included: 120 children who were hospitalised with HAdV pneumonia from June 2018 to December 2019, and 80 children with atopy and without HAdV infection who were selected as the control group. Based on the history of atopy, a total of 122 patients had atopy, both with and without HAdV infection, and 78 patients were non-atopic. There were 80 cases of mild pneumonia (30 atopic cases and 50 non-atopic cases) and 40 cases of severe pneumonia (12 atopic cases and 28 non-atopic cases). The 80 children with atopy and without HAdV infection were further divided into two groups according to disease severity (40 with mild pneumonia and 40 with severe pneumonia)

Based on the history of atopy, a total of 122 patients had atopy, both with and without HAdV infection, and 78 patients were non-atopic. There were 80 cases of mild pneumonia (30 atopic cases and 50 non-atopic cases) and 40 cases of severe pneumonia (12 atopic cases and 28 non-atopic cases). The 80 children with atopy and without HAdV infection were further divided into two groups according to pneumonia severity (40 with mild pneumonia and 40 with severe pneumonia; Fig. 1).

### Baseline and laboratory characteristics of children with HAdV infection and atopy and HAdV infection without atopy

Of the 200 children, 62 boys and 60 girls were included in the atopic group, and 45 boys and 33 girls were included in the non-atopic group. There was no difference in sex, age, neutrophil count, or lymphocyte count (*P* > 0.05) between the atopic and non-atopic groups. However, the eosinophil count in the atopic group was significantly higher than that in the non-atopic group (*P* < 0.05; Table 1).

**Table 1:** Baseline and laboratory characteristics of children with HAdV infection and atopy and HAdV infection without atopy

Group	Atopy (n = 122)	Non-atopy (n = 78)	X^2^/t-test	P value
Male	62 (50.8%)	45 (57.7%)	0.133	0.716
Female	60 (49.2%)	33 (42.3%)		
Age	5.2 ± 1.2	5.5 ± 0.6	1.230	0.34
Neutrophil count	(11.21 ± 0.03) × 10^9^/L	(11.80 ± 0.01) × 10^9^/L	1.280	1.050
Lymphocyte count	(2.74 ± 0.02) × 10^9^/L	(2.80 ± 0.13) × 10^9^/L	1.460	0.120
eosinophil count	(0.53 ± 0.01) × 10^9^/L	(0.22 ± 0.01) × 10^9^/L	1.829	0.020^*^
CRP	(36.27 ± 1.52) mg/L	(38.36 ± 2.69) mg/L	1.140	0.180

The bold values represent the factors that are significant differences between the two groups

^*^P < 0.05, compare with non-atopic group statistically significant. CRP: C-reactive protein;

### Differences in clinical characteristics between children with mild adenovirus infection with and without atopy

Considering the differences in clinical characteristics, the prognosis, and chest HRCT changes may due to pneumonia severity after infection, we analyzed the clinical characteristics of children with mild and severe pneumonia respectively. The numbers of children with mild pneumonia, severe cough, wheezing, and small airway lesions on chest HRCT upon admission and 1 month after discharge were significantly higher in the atopic group (with or without HAdV infection) than in the non-atopic group (*P* < 0.05). Additionally, the numbers of children in the atopic group with mild HAdV pneumonia, severe cough, wheezing, and small airway lesions on chest HRCT upon admission and 1 month after discharge were significantly higher than those in the atopic group without HAdV infection (*P* < 0.05). There was no significant difference in the duration of hospitalisation or fever among the three groups (*P* > 0.05; Table 2).

**Table 2:** Differences in clinical characteristics between children with mild adenovirus infection with and without atopy

Group	Atopic with HAdV (n = 30)	Non-atopic with HAdV (n = 50)	Atopic without HAdV (n = 40)
Clinic characteristics			
Duration of fever (day)	5.3 ± 1.1	5.5 ± 0.8	5 ± 1.2
Duration of hospitalization (day)	8.2 ± 1.5	8.1 ± 0.6	7 ± 1.0
Cases of wheezing in hospital (%)	11 (9.17%)^ab^	5 (4.17%)	4 (3.33%)^a^
Cases of wheezing after discharge (%)	5 (4.17%)^ab^	2 (1.67%)	3 (2.5%)^a^
Cases of severe cough during hospitalization	10 (8.33%)^ab^	7 (5.83%)	8 (6.67%)^a^
Pulmonary radiological changes			
Cases have small airway lesions (on admission)	6 (5%)^ab^	3 (2.5%)	4 (3.33%)^a^
Cases have small airway lesions (a month after discharge)	4 (3.33%)^ab^	2 (1.67%)	1 (0.83)^a^

^*a*^*P* < 0.05, compare with non-atopic with HAdV group statistically significant. ^b^*P* < 0.05, compare with atopic without HAdV group statistically significant

### Differences in clinical features and radiological changes between children with severe HAdV pneumonia with and without atopy

The numbers of cases of wheezing and small airway lesions during hospitalisation and after discharge were significantly higher in the atopic group (with or without HAdV infection) with severe pneumonia than in the non-atopic group (*P* < 0.05). There were significant differences in the number of patients with wheezing and small airway lesions during hospitalisation and after discharge between the atopic groups with and without HAdV infection (*P* < 0.05). A total of nine children with atopy were found with small airway lesions during hospitalisation (five cases, excessive permeability; three cases, mosaic sign; and one case, bronchial wall thickening). There were no significant differences in blood oxygen saturation, CRP levels, hospitalisation time, duration of fever, the presence of dyspnoea, and extra-pulmonary complications among the three groups (*P* > 0.05; Table 3).

**Table 3:** Differences in clinical features and radiological changes between children with severe HAdV pneumonia with and without atopy

Group	Atopic with HAdV (n = 12)	Non-atopic with HAdV (n = 28)	Atopic without HAdV (n = 40)
Clinical features			
Degree of blood oxygen saturation	(78.33 ± 2.35)%	(80.24 ± 3.25)%	(79 ± 2.16)%
Cases of dyspnea (%)	8 (10%)	6(7.5%)	7(8.75%)
Duration of fever (day)	7.1 ± 1.1	7.3 ± 0.8	7 ± 0.9
Duration of hospitalization (day)	9.2 ± 1.5	9.3 ± 0.6	9 ± 1.3
Cases of wheezing in hospital (%)	8 (10%)^ab^	4 (5%)	6 (7.5%)^a^
Cases of wheezing after discharge (%)	6 (7.5%)^ab^	2 (2.5%)	4 (5%)^a^
Cases of extra-pulmonary complications (%)	16 (20%)	15 (18.75%)	18 (22.5%)
Pulmonary radiological changes			
Cases have small airway lesions (on admission)	9 (11.25%)^ab^	5 (6.25%)	7 (8.75%)^a^
Cases have small airway lesions (a month after discharge)	4 (5%)^ab^	1 (1.25%)	3 (3.75%)^a^

^*a*^*P* < 0.05, compare with non-atopic with HAdV group statistically significant. ^b^*P* < 0.05, compare with atopic without HAdV group statistically significant

### Risk factors associated with post-HAdV pneumonia with small airway lesions

As small airway lesion is the most common complication of post- HAdV infection, we compared the baseline characteristics and symptoms of the children with pneumonia with and without small airway lesions to identify the risk factors of small airway lesion. According to the imaging findings, 200 children were divided into two groups according to the presence or absence of small airway lesions. We found that in the small airway lesions group, the numbers of patients with atopy, severe infection, and a family or personal history of asthma were significantly higher than those in the non-small airway lesion group (*P* < 0.05; Table 4). We also performed a multivariate analysis to identify risk factors associated with small airway lesions in patients with HAdV pneumonia. We found that a family history of asthma (OR 2.1 [95% CI 1.8–3.0]), personal history of asthma (OR 2.7 [95% CI 2.1–3.1]), atopy (OR 2.1 [95% CI 1.8–3.2]), severe infection (OR 1.9 [95% CI 1.0–2.7]), and HAdV infection (OR 1.4, [95% CI 0.9–2.0]) were independent factors associated with the development of small airway lesions, based on chest HRCT findings (Table 5).

**Table 4:** Baseline characteristics, symptoms of the 200 children with pneumonia with and without small airway lesions

Characteristics	Total (n = 200) n (%)	With small airway lesions (n = 34) n (%)	Without small airway lesions (n = 166) n (%)	X^2^/t-test	P value
Atopy				6.556	0.01^*^
Yes	122 (61%)	26 (76.5%)	96 (59.6%)		
No	78 (39%)	8 (23.5%)	69 (41.4%)		
Severe infection				13.975	0.01^*^
Yes	80 (40%)	21 (61.8%)	59 (35.5%)		
No	120 (60%)	13 (38.2%)	107 (64.5%)		
Fever				0.02	0.887
Yes	110 (55%)	16 (47.1%)	80 (48.2%)		
No	90 (45%)	18 (52.9%)	86 (51.8%)		
Duration of hospitalization		8.2 ± 1.1	8.3 ± 0.7	0.984	0.06
Family history of asthma				35.498	0.001*
Yes	50 (25%)	20 (58.8%)	30 (18.1%)		
No	150 (75%)	14 (41.2%)	136 (81.9%)		
Previous chest infection				0.041	0.84
Yes	55 (27.5%)	9 (26.5%)	46 (27.7%)		
No	145 (72.5%)	25 (73.5%)	120 (72.3%)		
Passive smoking				0.471	0.493
Yes	54 (27%)	8 (23.5%)	46 (27.7%)		
No	146 (73%)	26 (76.5%)	120 (72.3%)		
Personal history of asthma/wheeze				31.165	0.001*
Yes	81 (67.5%)	25 (73.5%)	56 (33.7%)		
No	119 (23.5%)	9 (26.5%)	110 (66.3%)		
HAdV infection				5.937	0.015*
Yes	120 (60%)	23 (67.6%)	97 (58.4%)		
No	80 (40%)	11 (23.4%)	69 (41.6%)		

The bold values represent the factors that are significant differences between the two groups

^*^P < 0.05, compare with the group without small airway lesions statistically significant

**Table 5:** Multivariate analysis of risk factors associated with post-adenovirus pneumonia with small airway lesions

Characteristics	Total (n = 200) n (%)	With small airway lesions (n = 34) n (%)	Without small airway lesions (n = 166) n (%)	P value	Odds ratio 95% (confidence interval)
Atopy	122 (61%)	26 (76.5%)	96 (59.6%)	0.021*	2.1 (1.8–3.2)
Severe infection	80 (40%)	21 (61.8%)	59 (35.5%)	0.020*	1.9 (1.0–2.7)
Family history of asthma	50 (25%)	20 (58.8%)	30 (18.1%)	0.019*	2.4 (1.8–3.0)
Personal history of asthma/wheeze	81 (67.5%)	25 (73.5%)	56 (33.7%)	0.018*	2.7 (2.1–3.1)
HAdV infection	120 (60%)	23 (67.6%)	97 (58.4%)	0.023*	1.4 (0.9–2.2)

^*^P < 0.05, compare with the group without small airway diseases statistically significant

## Discussion

Children with atopy are a special population, and atopy is often accompanied by airway hyperresponsiveness. In our study, we compared the clinical characteristics of children with atopy and HAdV pneumonia to children without atopy. We found that there was no difference in the duration of fever between the two groups. However, the eosinophil count in the atopic group was significantly higher than that in the non-atopic group, which was considered to be related to the fact that the children not allergens avoidance recently, because not all of the children in the atopic group had increased eosinophil count in blood. The eosinophil count in part of the children with atopy without HAdV infection is also higher, so we considered the higher eosinophil count not due to HAdV infection, but atopy. Considering the differences in clinical features and chest HRCT changes due to pneumonia severity after infection, we analyzed the clinical characteristics of children with mild and severe pneumonia respectively. In children with atopy (with or without HAdV infection) and mild pneumonia, the number of cases of severe cough or wheezing was significantly higher than that in those without atopy, and the number of patients with wheezing during hospitalisation was significantly different. Therefore, we concluded that after HAdV infection, children with atopy are more prone to severe coughing and wheezing than children without atopy. The reason for wheezing in children with atopy and HAdV pneumonia may be related to the exposure of the damaged airway epithelium neurons and increased airway sensitivity [18]. Studies have shown that leukotriene E4 is strongly associated with episodes of acute wheezing in preschool children and that the levels of leukotriene E4 are higher in the airways of children with atopy than in those of children without atopy [19]. In our study, we also found that the number of cases of wheezing in the atopic group with severe HAdV pneumonia was significantly higher than that in the atopic group without HAdV infection. The reason may be related to the characteristics of HAdV infection. Firstly, damage to the airway mucosa after HAdV infection and the release of inflammatory mediators can cause mucosal oedema of the bronchi and bronchioles, congestion, necrosis and shedding, necrotic obstruction of the lumen, and bronchial wall oedema and thickening, resulting in vasospasm and muscle contraction. As airway epithelial cells are damaged, their defence capacity is reduced, resulting in allergens invading the airway more easily [12]. In contrast, toll-like receptors (TLR) and intracellular virus sensors such as protein-catalysed enzymes (protein kinase double-stranded RNA, PKR) in airway epithelial cells [13] induce MUC5AC production, leading to airway epithelial mucus hypersecretion and blockage of the lumen. After infection, HAdV can interact with the host cells and extensively participate in the functions of host cell proliferation, apoptosis, autophagy, and so on [18]. Studies have shown that when HAdV infects respiratory epithelial cells, host cells develop adaptive autophagy, which enables immune evasion [19], leading to autophagy dysregulation in the host cells. HAdV can induce the activation of CD8^+^ T cells through the autophagy pathway, leading to microenvironmental changes in the lung tissue [20]. In the airways of children with atopy, damaged neurons in the airway epithelia are exposed, increasing airway sensitivity [21] and leading to more serious epithelial injury, thus making patients more susceptible to wheezing.

The changes caused by HAdV pneumonia on the chest CT image include lung consolidation, patchy shadows, flocculent shadows, cluster shadows, air bronchograms, and lymph node enlargement. Its effects on the small airways include uneven inflation, mosaic sign, bronchial thickening, and bronchiectasis [20]. In the current study, we compared children with HAdV pneumonia with and without atopy and found that the percentage of children with atopy and small airway lesions was more than that of children without atopy. We also compared the baseline characteristics and symptoms of children with pneumonia with and without small airway lesions. We found that in the small airway lesions group, the numbers of patients with atopy, severe infection, and a family or personal history of asthma were significantly higher than those in the group without small airway lesions. A family or personal history of asthma, atopy, severe infection, and HAdV infection were independent factors associated with the development of small airway lesions, based on chest HRCT findings.

Uneven inflation of the lungs is always found on chest CT in children with atopy, and children often experience coughing or wheezing which requires treatment after discharge. The changes in the lung parenchyma, such as lung consolidation, usually recover slowly after discharge and most small airway lesions require atomisation to recover; however, we observed that, even after 1 month, some children with atopy had small airway lesions on the chest CT. Some children were still intolerant to sports activities, had post-activity wheezing, and showed progression to bronchiolitis obliterans.

The small airway refers to the airway with an inner diameter ≤ 2 mm and is one of the smallest visible areas of the lungs. Most of the airways are referred to as bronchioles, belonging to the 12th–23rd branch of the airway [21]. There are direct and indirect manifestations of small airway lesions on HRCT. The direct signs, including central lobular nodules, tree bud signs, and bronchiectasis, are caused by thickening of the bronchial wall or bronchiectasis [22]. Indirect signs are caused by the obstruction of bronchioles and include mosaic sign and gas trapping [23]. HRCT imaging of small airway lesions can detect the following: thickening of the bronchiole wall; tree bud sign; mosaic characteristics; and air retention. The mechanism for the development of small airway lesions in children with atopy may be eosinophilia and an abundance of CD4^+^ T lymphocytes in the small airways compared to that in the larger airways, which results in small airway inflammation [24]. Small airway inflammation, airway remodelling, and matrix deposition eventually lead to increased airway resistance, similar to the pathophysiologic changes that occur in the small airways of patients with asthma [25]. In our study, we found that the number of patients with small airway lesions during hospitalisation and after discharge was significantly higher in the atopic group with severe HAdV pneumonia than in the atopic group without HAdV infection. The reason may be that small airway lesions are also associated with persistent latent infection, attributed to the HAdV E1A genes. The adenovirus genome comprises linear double-stranded DNA, containing five early transcription units, namely E1A, E1B, E2, E3, and E4. The viral genome translocates to the host nucleus, and its transcription and expression initiate and facilitate viral replication. E1A is the earliest transcribed gene [26]. Studies have demonstrated that the adenoviral E1A DNA and proteins persist in the lung tissue after viral replication stops in the acute infection phase; this enables the long-term expression of proteins without the need for replication of the entire virus. The main target cells are bronchial epithelial cells, alveolar epithelial cells, and submucosal cells [27]. Studies have shown that enhanced expression of E1A genes can activate the mitogen-activated protein kinase (MAPK) signalling pathway, allowing HAdV to proliferate continuously in respiratory epithelial cells [24]. Persistent latent infection of the lung tissue by E1A genes may lead to airway remodelling [28].

There are many limitations to our study. We only considered the influence of atopy on the clinical symptoms of children, and other confounding factors that may also affect the prognosis of children, such as co-infection with respiratory syncytial virus [28], mycoplasma infection, influenza [29], and mixed influenza infections, were not considered. In some patients with wheezing, the use of glucocorticoids to suppress immune responses may have also affected the prognosis of pneumonia, which was not considered in this study. The difference in the timing of treatment and virus typing in some patients with severe pneumonia may also affect their prognosis, which was not considered in this study [2]. Therefore, our center will carry out relevant cohort studies in the future.

## Conclusion

Children with atopy and HAdV pneumonia may experience severe coughing in mild cases and wheezing in mild and severe cases. Children with atopy are more susceptible to the development of small airway lesions, recurrent wheezing after discharge and slower recovery of small airway lesions as observed on pulmonary imaging than non-atopic children after HAdV infection. A family or personal history of asthma, atopy, severe infection, and HAdV infection are independent risk factors associated with the development of small airway lesion as observed on chest HRCT.

## Data Availability

The datasets used and/or analysed during the current study are available from the corresponding author on reasonable request.

## Abbreviations

HAdV: Adenovirus
HRCT: High-resolution computer tomography
CRP: C-reactive protein
MAPK: Mitogen-activated protein kinase
TLR: Toll-like receptors
